# *In vitro* potency of amikacin against carbapenem-resistant Pseudomonas aeruginosa: A target for nebulization strategy?

**DOI:** 10.1016/j.bjid.2022.102355

**Published:** 2022-04-28

**Authors:** Gabriel T. Cuba, Paulo H.D. Santos, Antonio C.C. Pignatari, David P. Nicolau, Carlos R.V. Kiffer

**Affiliations:** a Universidade Federal de São Paulo (UNIFESP), Escola Paulista de Medicina, São Paulo, SP, Brazil; b Universidade de São Paulo (HCFMUSP), Hospital das Clínicas, Faculdade de Medicina, São Paulo, SP, Brazil; c Center of Anti-infective Research and Development, Hartford Hospital, Hartford, CT, United States

Dear Editor,

Pharmacodynamic model simulating amikacin epithelial lining fluid exposures showed that inhaled amikacin monotherapy provided bactericidal activity against isolates tested at MICs ≤ 256 mg/L.^1^ Then, we decided to investigate the *in vitro* potency of amikacin against Carbapenem-Resistant *P. Aeruginosa* (CRPA) isolates representative from Brazilian ICU settings. Seventy-one (71) non-duplicate isolates of *P. aeruginosa* from 10 different ICUs from Brazil were included.^2^ *P. aeruginosa* were recovered from nosocomial bloodstream (*n* = 47, 66.2%) and respiratory tract (*n* = 24, 33.8%). Approximately two-thirds of the patients were male (*n* = 47, 66.2%) and had a mean age of 57 ± 18.2 years (range, 5 to 83 years).

The presence of 16S RNA methyltransferase-encoding genes (armA, rmtD, and rmtB) was evaluated by Real Time Polimerase Chain Reaction (RT-PCR) using the Rotor-Gene Q (Qiagen, Germany) and High-Resolution Melt-HRM master mix (HRM PCR buffer type, EvaGreen dye, Qiagen, Germany). The identification was performed by multiplex RT-PCR using specific primers of each gene (armA, rmtB and rmtD) as previously described.^3^ The detection of metallo-beta-lactamase-enconding-genes bla_SPM-1_, bla_IMP_ and bla_VIM_ was performed by a Multiplex Real Time PCR as previously described. The presence of bla_SPM-1_ was confirmed by a single RT-PCR using the primer pair previously described by Mendes and colleagues.^4^

The Susceptibility Testing (AST) for amikacin (Sigma-Aldrich, St. Louis, USA) was performed by agar dilution and the results were interpreted according to Clinical and Laboratory Standards Institute (CLSI),^3^^,^^5^^,^^6^ with amikacin concentration ranging from 0.25 to > 4096 mg/L and meropenem from 16 to 512 mg/L. Isolates with amikacin MIC > 16 mg/L were selected for 16S RNA methyltransferase and metallo-beta-lactamase testing by polymerase chain reaction.^4^^,^^7^

MIC by AD distribution for amikacin and meropenem is shown in Fig. 1. Forty-seven isolates with amikacin MIC > 16 mg/L were submitted to 16S RNA methyltransferase-encoding and metallo-beta-lactamase-enconding gene detection. Forty-one (87.2%) isolates presented rmtD. Twenty-one (21/47; 44.7%) of those isolates presented bla_SPM-1_. Amikacin MICs ≥ 4,096 mg/L was found among 19/21 isolates co-producing bla_SPM-1_ and rmtD genes and among only 9/26 isolates bla_SPM-1_ negative and rmtD positive. Higher meropenem MICs (64 to 512 mg/L) were observed more commonly among SPM-1-producers isolates (16/22; 72.7%) while 80% (20/25) of the isolates with lower meropenem MICs (16 to 32 mg/L) did not produce any metallo-beta-lactamase. Higher meropenem MICs were already associated with metallo-beta-lactamase production, including SPM-1.^7^

**Fig. 1:**
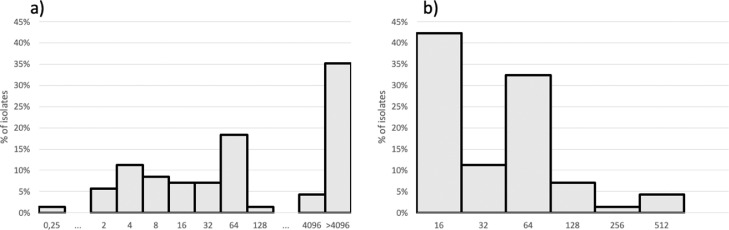
Distribution of MIC frequency of Amikacin (a) and Meropenem (b) against carbapenem-resistant *P. aeruginosa*.

For patients with VAP due to Gram-negative bacilli that are susceptible to only last resort drugs, adjunctive inhaled antibiotic associated with systemic antibiotics could be prescribed. However, aminoglycoside monotherapy is not recommended to be used to treat patients with HAP/VAP due to *P. aeruginosa*.^8^ This study aimed at understanding the amikacin potency against clinically significant and resistant *P. aeruginosa*, representing difficult-to-treat respiratory infections with antimicrobial concentrations virtually unachievable by standard intravenous treatments. Approximately two thirds (61%) of those isolates included in this collection had MICs ≤ 256 mg/L and thus may be suitable to treatment using amikacin via the nebulization route. Nonetheless, 16S rRNA methyltransferase RmtD dissemination throughout Brazil associated to SPM production narrows the potential usefulness of aminoglycosides as a treatment option for salvage therapy of VAP and HAP caused by carbapenem-resistant *P. aeruginosa*.

## Conflicts of interest

The authors declare no conflicts of interest.
